# A111 CHARACTERISTICS OF HOSPITALIZED PATIENTS WITH GASTROINTESTINAL DISEASE WHO LEAVE AGAINST MEDICAL ADVICE

**DOI:** 10.1093/jcag/gwad061.111

**Published:** 2024-02-14

**Authors:** A Rivas, E Purdy, K Buhler, J Flemming, C Ma

**Affiliations:** University of Calgary Cumming School of Medicine, Calgary, AB, Canada; Queen's University, Kingston, ON, Canada; University of Calgary Cumming School of Medicine, Calgary, AB, Canada; Queen's University, Kingston, ON, Canada; University of Calgary Cumming School of Medicine, Calgary, AB, Canada

## Abstract

**Background:**

Hospitalized inpatients who leave against medical advice (AMA) may have incomplete care, resulting in readmission and increased healthcare costs. Limited evidence exists in patients diagnosed with a primary gastrointestinal (GI) disease who leave AMA. Identifying patients with GI diagnoses who leave AMA may inform risk stratification to determine which patients are at risk of unplanned discharge.

**Aims:**

Identify characteristics in patients diagnosed with GI diseases who leave AMA.

**Methods:**

A retrospective evaluation was conducted using the National Inpatient Sample (NIS) from 2016 to 2020. The NIS provides national-level estimates of cost, quality, and outcomes from hospitalizations in the United States. We used International Classification of Diseases, Tenth Revision diagnostic coding to identify patients admitted with a primary GI problem. The proportion of patients who left AMA for each diagnosis was determined (Figure 1). A multivariable logistic regression was performed to determine factors associated with an AMA discharge, adjusting for age, sex, elective or weekend admission, primary payment method, race, hospital setting, geography, income, Elixhauser comorbidity index, alcohol and/or drug abuse and depression status.

**Results:**

Between 2016 and 2020, 1.61% of patients with a GI primary problem left the hospital AMA. Characteristics associated with leaving AMA included: older age, female sex, non-white race, rural hospital admission, higher income and having comorbid conditions (Table 1). Patients with a history of alcohol abuse were more likely to leave AMA, while patients with a history of drug abuse were less likely to leave AMA.

**Conclusions:**

Approximately 1 in 60 patients admitted with a primary GI problem leave hospital AMA and we identify both structural and patient-level factors associated with unplanned discharge.

Table 1: Factors Associated With AMA Discharge For GI-related Diagnoses

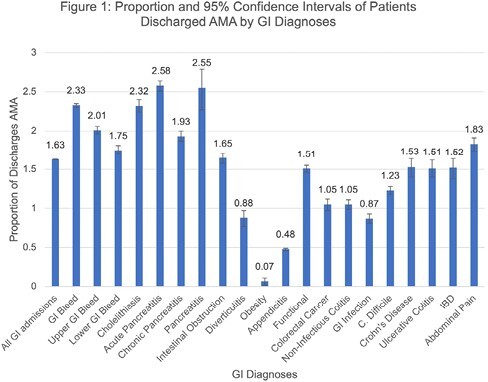

**Funding Agencies:**

None

**Table:** 

Characteristics	Multivariable Adjusted OR [95% CI]	p-value
Age: 18-34 years 35-64 years ≥65 years	- Reference 1.06 [1.02 – 1.11] 1.23 [1.17 – 1.30]	- 0.004 ampersand:003C0.001
Female sex	0.79 [0.77 – 0.81]	ampersand:003C0.001
Elective admission	0.46 [0.44 – 0.49]	ampersand:003C0.001
Weekend admission	1.03 [1.01 – 1.06]	0.011
Primary method of payment: Medicare Medicaid Private Insurance Other	- Reference 0.97 [0.93 – 1.01] 0.94 [0.90 – 0.98] 0.92 [0.87 – 0.97]	- - 0.157 0.001 0.002
Race: White Black Hispanic Other	- Reference 0.77 [0.73 – 0.80] 0.71 [0.68 – 0.75] 0.89 [0.84 – 0.94]	- - ampersand:003C0.001 ampersand:003C0.001 ampersand:003C0.001
Hospital Setting: Rural Urban Non-teaching Urban teaching	- Reference 0.55 [0.53 – 0.58] 0.30 [0.29 – 0.32]	- - ampersand:003C0.001 ampersand:003C0.001
Geography: Northeast Midwest South West	- Reference 1.02 [0.97 – 1.08] 0.75 [0.71 – 0.78] 0.88 [0.83 – 0.93]	- - 0.488 ampersand:003C0.001 ampersand:003C0.001
Income: 0-25% 25-50% 50-75% 75-100%	- Reference 1.00 [0.96 – 1.03] 0.85 [0.82 – 0.88] 0.76 [0.73 – 0.80]	- - 0.822 ampersand:003C0.001 ampersand:003C0.001
Comorbidity: Elixhauser Index = 0 Elixhauser Index = 1 Elixhauser Index ampersand:003E1	- Reference 1.10 [1.04 – 1.15] 1.46 [1.39 – 1.53]	- - ampersand:003C0.001 ampersand:003C0.001
Alcohol Abuse	1.22 [1.17 – 1.26]	ampersand:003C0.001
Drug Abuse	0.75 [0.70 – 0.79]	ampersand:003C0.001
Depression	0.85 [0.82 – 0.88]	ampersand:003C0.001

